# Long-term stability of the urogenital microbiota of asymptomatic European women

**DOI:** 10.1186/s12866-021-02123-3

**Published:** 2021-02-25

**Authors:** Magdalena Ksiezarek, Svetlana Ugarcina-Perovic, Joana Rocha, Filipa Grosso, Luísa Peixe

**Affiliations:** UCIBIO-REQUIMTE. Laboratory of Microbiology, Faculty of Pharmacy, University of Porto, Porto, Portugal

**Keywords:** Microbiome, Culturomics, Species diversity, Uropathogens, Voided midstream urine

## Abstract

**Background:**

To date, information on healthy female urinary microbiota is available mostly at genus level and at one time point. However, profound species-level characterization of healthy urinary microbiome and its stability over time are essential for further correct interpretation of its role in healthy urogenital tract. In this study, we investigated female urogenital microbiome (FUM) at two timepoints (within 2.5-year interval) in young asymptomatic European women. We used culturomics with accurate isolates’ identification (MALDI-TOF MS and gene markers sequencing) to understand species stability within healthy FUM.

**Results:**

Extended culturomics of voided midstream urine sample pairs revealed a mean Shannon diversity index of 1.25 and mean of 19 species/sample (range 5–39 species; total of 115 species; 1830 isolates). High overall species variability between individuals was captured by beta diversity and a variety of community structure types, with the largest cluster characterized by *Lactobacillus crispatus*, often in combination with *Gardnerella vaginalis* or *Gardnerella* genomospecies 3. Significant FUM composition differences, related to *Finegoldia magna* and *Streptococcus anginosus,* according to smoking status were found.

A high species variability within individuals (Shannon index SD > 0.5 in 7 out of 10 sample pairs) with a mean of 29% of shared species (range 9.1–41.7%) was observed. Moreover, 4 out of 10 sample pairs clustered in the same community structure type. The stable FUM sample pairs presented high abundance of *Lactobacillus crispatus, Streptococcus agalactiae* or *Lactobacillus paragasseri* and *Bifidobacterium* spp.. Moreover, *Gardnerella vaginalis*, *Gardnerella* genomospecies 3 or *Gardnerella swidsinskii* were often maintained within individuals in high abundance.

**Conclusions:**

Shift in species composition at two distant timepoints was frequently observed among urogenital microbiome of European asymptomatic women. This suggests possible interchange of particular species in healthy FUM and the existence of multiple health-associated FUM compositions in certain individuals.

Additionally, we provided additional evidence on resilience of particular bacterial communities and identified certain species more prone to persist in urogenital tract.

This study revealed important details on the FUM composition complexity relevant for studies aiming to understand microbiota role in the urogenital tract health and for identification of eubiotic and dysbiotic FUM.

**Supplementary Information:**

The online version contains supplementary material available at 10.1186/s12866-021-02123-3.

## Background

In the recent years, novel high-throughput culture- and DNA-based studies revealed the existence of a microbial community inhabiting the human lower urinary tract [1–8]. The majority of available observations have been made in female urogenital microbiota (FUM) composition under disease state and, simultaneously, data originated from asymptomatic controls provided a broad overview at high taxonomic levels on healthy FUM [2, 6, 9–11].

To date, a set of microbiota profiles based on dominant taxa, with interpersonal differences in bacterial load, diversity and abundance of specific bacteria, has been reported. *Lactobacillus*, *Gardnerella* and *Streptococcus* genera have been often highlighted as most prevalent healthy FUM members, in combinations with other genera such as *Staphylococcus, Corynebacterium* or *Escherichia* [2, 12–16]. A few studies indicated species such as *Lactobacillus crispatus*, *Lactobacillus jensenii, Gardnerella vaginalis* or *Streptococcus anginosus*, identified by MALDI-TOF MS analysis, as the most prevalent within healthy FUM [2, 12, 16, 17].

Since it is widely recognized in other body niches that microbiota dysbiosis, i.e., significant change in microbiota composition, may contribute to disease development [18, 19], it is important to assess the scale of microbiota compositional shifts occurring naturally in healthy urogenital tract and evaluate resilience of urogenital microbiota. To date, three-months daily assessment of female urinary microbiota demonstrated that it can be both dynamic and resilient. Moreover, changes in urinary microbiota composition may occur daily and certain shifts are associated with particular physiological or lifestyle factors, such as increased detection of *Streptococcus* and *Staphylococcus* genus associated with vaginal intercourse, or increased detection of e.g., *Corynebacterium* and *Actinomyces* during menstruation [14].

Therefore, fundamental knowledge on urogenital microbiota compositional stability remains incomplete and needs to be enlarged by long-term studies, addressing adequately the species shifts occurring in the urogenital tract and preferably eliminating factors known to alter microbiota structure.

To evaluate compositional stability of healthy FUM at two timepoints within a long period of time (2.5-year interval), we performed a comprehensive culturomic-based analysis (extended number of characterized isolates and improved methodologies for bacterial identification) of voided midstream urine samples of ten reproductive-age asymptomatic women. To the best of our knowledge, this is the first study assessing long-term FUM compositional stability at two distant timepoints in urogenital tract of asymptomatic women.

## Results

### Cohort overview

Ten asymptomatic reproductive-age women (24–40 years old) provided voided midstream urine samples (*n* = 20) at two time points within the 2.5-year interval. All participants were residents of Portugal, declared to have a balanced diet, and reported good or very good general health conditions according to their individual interpretation. None of the participants had symptoms or discomfort associated with their urogenital tract at either sampling time. Although some participants declared to have had UTI in the past, none of them reported to suffer from recurrent UTIs. Additionally, 2 participants acquired UTI (U7, U23) in the time between first and second sampling. One participant resigned from hormonal contraception (U4) in the interval between samplings. Three individuals reported themselves as active smokers (U9, U15, U26). Detailed demographic information about participants at the first and second sampling time is provided in Table 1.

**Table 1:** Demographic characteristics of participants

METRIC	First sampling	Second sampling
Age (years)	mean = 30.7 (SD = 4.97)	mean = 32.5 (SD = 4.97)
BMI (kg/m^2^)	mean = 21.74 (SD = 2.18)	mean = 21.76 (SD = 2.24)
Smokers	30%	30%
Sexually active	100%	100%
Regular menstrual cycle	90%	90%
Previous pregnancy	40%	40%
Hormonal contraception	90%	80%
Anti-inflammatory drugs usage in week before sampling	30%	20%
UTI in the past	40%	60%

Age and BMI expressed in mean and standard deviation (SD). Remaining parameters expressed in % of positive women

### Culturomic analysis overview

The bacterial load varied from 10^^^4 to 10^^^8 CFU/ml with maximum difference of 10^^^2 CFU/ml for sample pair (4 out of 10 sample pairs). A range of 17–321 (mean = 103, median = 63) isolates per sample was characterized. Identification of 1830 bacterial isolates resulted in detection of 5 phyla, 48 genera and 115 species. Overall, identified taxa distribution at phylum level was characterized by dominance of the Firmicutes (50–51% of total species for first and second sampling, respectively) and Actinobacteria (40%; 30%), and less prevalent Proteobacteria (6%; 11%), Bacteroidetes (3%; 3%) and Fusobacteria (1%; 2%). A list of species detected in each participant during first and second sampling can be found in Additional file 1: Table S1.

### Diversity of healthy FUM over time

**Fig. 1:**
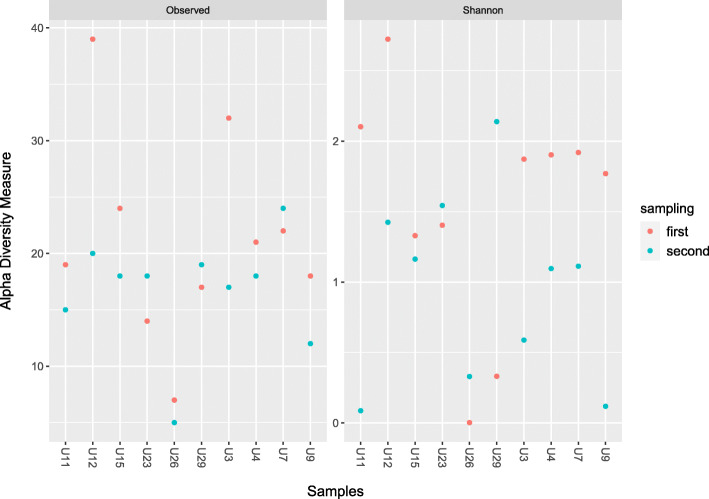
Alpha diversity among samples measured by observed number of species and Shannon index

Overall, alpha diversity represented by mean Shannon index was 1.25 (standard deviation = 0.79; SD), species richness varied within range of 5 to 39 species/sample (mean = 19, SD = 8) and species evenness (Pielou’s evenness index) varied within range of 0.0002 to 0.29 per sample (mean = 0.18, SD = 0.1). All values of species richness, evenness and Shannon index per each sample are presented in Additional file 1: Table S2. Species richness for sample pairs differed in a range of 2 to 18 species. Shannon index SD for sample pairs was ranging from 0.12 to 1.44. In 7 out of 10 sample pairs Shannon index SD was higher than 0.5. Graphic representation of alpha diversity measures is presented in Fig. 1.

**Fig. 2:**
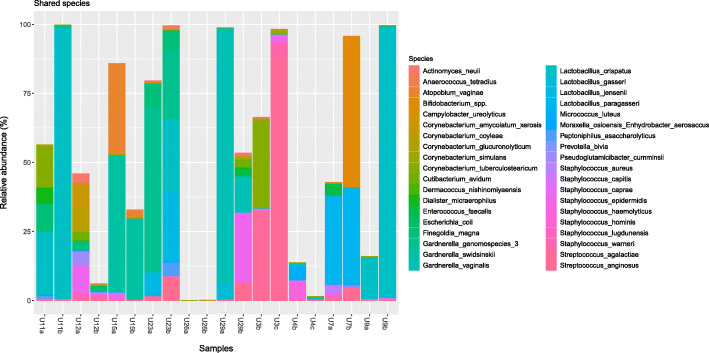
Summarized relative abundance (%) of shared species in each sample. Sample pairs are ordered next to each other

Sample pairs presented a range of 1–12 (median of 10) species in common. Percentage of shared species within individual over time was in a range of 9.1–41.7% (mean = 29%), with changes in their relative abundance (Fig. 2). Species observed in both samples of at least one participant, corresponded to 38 out of 115 species detected. Those included prevalent *Staphylococcus epidermidis, Micrococcus luteus, Streptococcus anginosus* and *Staphylococcus haemolyticus* (in more than 5 sample pairs) mostly present in low relative abundance. Additionally, *Lactobacillus crispatus, Gardnerella vaginalis, Gardnerella swidsinskii, Gardnerella* genomospecies 3 and *Streptococcus agalactiae* were among shared species however usually present in high relative abundance (range of 29–44% average relative abundance).

**Fig. 3:**
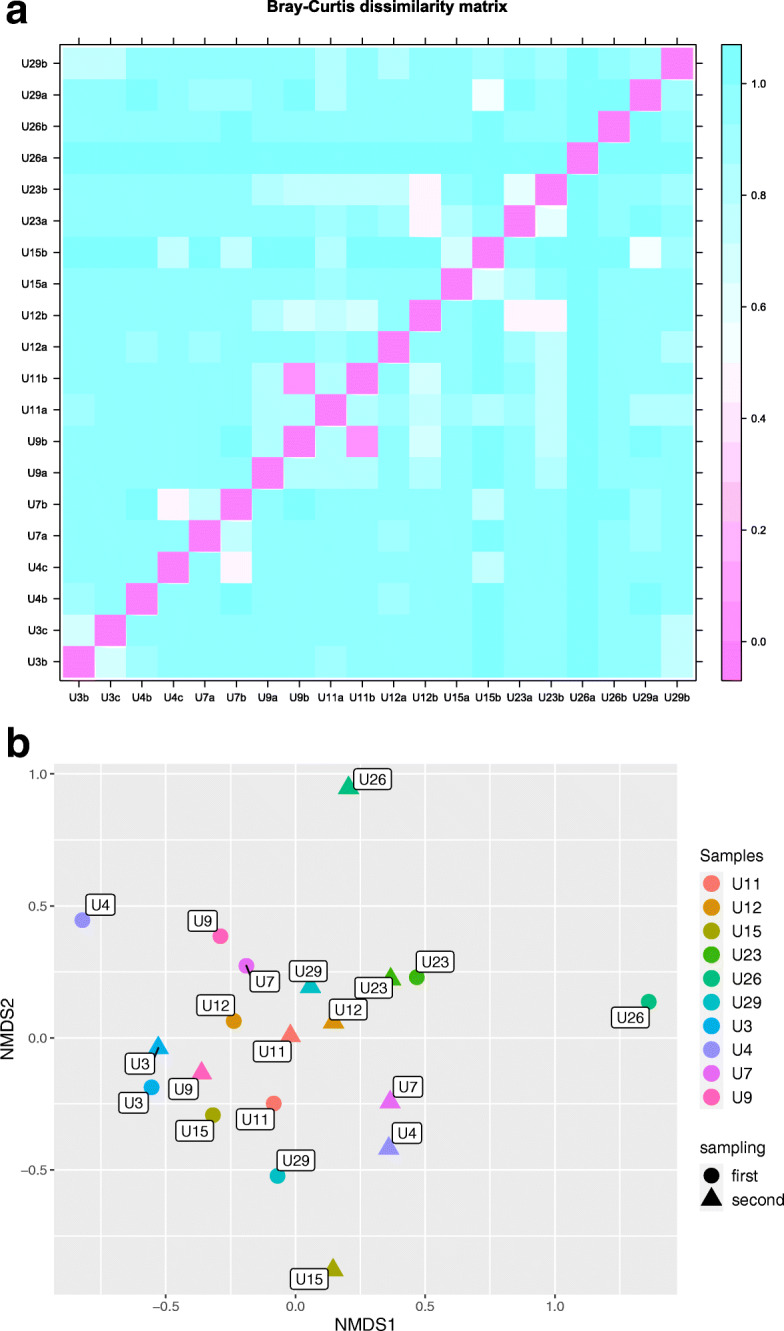
Beta diversity among samples. **a** Heatmap representing Bray-Curtis dissimilarity matrix between the samples. **b** Relationship between samples presented by 2-dimension NMDS ordination based on Bray-Curtis distance matrix, with 0.2 stress value

Beta diversity is presented with Bray-Curtis dissimilarity matrix (Fig. 3a) and two-dimension non-metric ordination (Fig. 3b). Most samples between individuals (6/10) were different based on Bray-Curtis dissimilarity > 0.5. In NMDS ordination (stress value 0.2) U26 sample pair was observed as the more dissimilar pair and was previously characterized by particularly low species richness. ANOSIM test revealed statistically significant differences between bacterial communities and smoking status of the individuals (R = 0.25, *p* = 0.03). Multilevel pattern analysis identified 2 species associated with smoking status and FUM variance, namely *Finegoldia magna* (*p* < 0.05) and *Streptococcus anginosus* (p < 0.05). Remaining factors tested did not show statistically significant microbiota composition differences.

### Healthy FUM community structure types over time

**Fig. 4:**
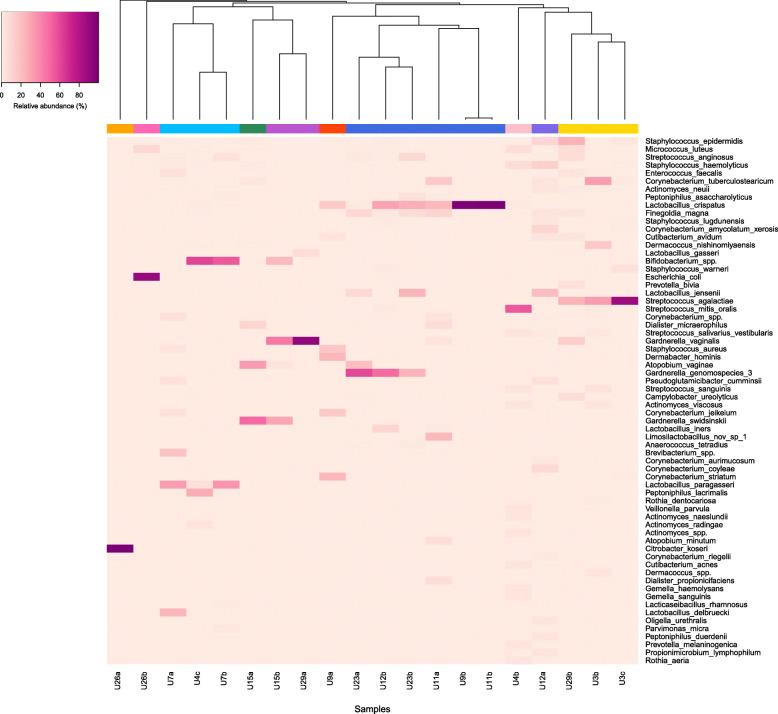
Heatmap based on abundance (%) of species detected at least once in abundance more than 1%. Species are ordered in decreasing prevalence. Dendrogram presents hierarchical clustering of microbiota profiles into community structure types, based on 0.8 cutoff. Colorful bar below the dendrogram stands for different community structure types

FUM structure types identified in asymptomatic women are presented in Fig. 4. Ten community structure types (dendrogram representing samples hierarchical clustering based on species level identification is available in Additional file 2: Fig. S1) were identified. The largest cluster (6 out of 20 samples) was characterized by *Lactobacillus crispatus,* often in combination with *Gardnerella* spp. namely*, Gardnerella vaginalis* or *Gardnerella* genomospecies 3. The other more common clusters presented abundant *Streptococcus agalactiae* or abundant *Bifidobacterium* spp. and *Lactobacillus paragasseri*, with other Gram-positive bacteria in lower abundance. Summary of clusters with different bacterial combinations characterizing community structure types are presented in Table 2.

**Table 2:** Summary of community structure types detected within healthy FUM

Community structure type	Characteristic species combination (ordered by decreasing relative abundance, only top 3 shown)	Samples	Shannon index (mean, SD – standard deviation)
1	*Citrobacter koseri* *Enterococcus faecalis* *Lactobacillus jensenii*	U26a	0.002
2	*Escherichia coli* *Micrococcus luteus* *Lactobacillus jensenii*	U26b	0.33
3	*Bifidobacterium* spp. *Lactobacillus paragasseri* *Enterococcus faecalis*	U4c U7a, U7b	1.38 (SD 0.47)
4	*Gardnerella swidsinskii* *Atopobium vaginae* *Dialister microaerophilus*	U15a	1.33
5	*Gardnerella vaginalis* *Bifidobacterium* spp. *Cutibacterium avidum*	U15b U29a	0.75 (SD 0.59)
6	*Corynebacterium striatum* *Dermabacter hominis* *Staphylococcus aureus*	U9a	1.77
7	*Lactobacillus crispatus*	U9b U11a, U11b U12b U23a, U23b	1.11 (SD 0.84)
8	*Streptococcus mitis/oralis* *Staphylococcus haemolyticus* *Micrococcus luteus*	U4b	1.90
9	*Lactobacillus jensenii* *Staphylococcus haemolyticus* *Staphylococcus epidermidis*	U12a	2.72
10	*Streptococcus agalactiae* *Staphylococcus epidermidis* *Corynebacterium tuberculostearicum*	U3b, U3c U29b	1.53 (SD 0.83)

*Gardnerella vaginalis* and the recently described *Gardnerella* species (*Gardnerella swidsinskii* or *Gardnerella* genomospecies 3) were observed in 5 individuals, usually with single species per individual (4/5) and *Gardnerella vaginalis* was the more prevalent one. Moreover, the recently described *Lactobacillus mulieris*, originally isolated from other cohort of our FUM study [20], was also identified in one individual (U26b). Furthermore, two putatively new species close to *Limosilactobacillus vaginalis* were also depicted in 2 individuals (U9a and U11a) (data not shown).

Different community structure types were observed within 6 out of 10 sample pairs, with changes related to genus or species presence and abundance [e.g., *Lactobacillus jensennii, Staphylococcus haemolyticus, Staphylococcus epidermidis* type (U12a) converted to *Lactobacillus crispatus* type (U12b) or *Gardnerella swidsinskii, Atopobium vaginae* and *Dialister microaerophilus* type (U15a) converted to *Gardnerella vaginalis*, *Bifidobacterium* spp., *Cutibacterium avidum* community structure type (U15b)]. Noteworthy, an individual with highly abundant Enterobacteriaceae members (U26) presented a shift in the community structure type (*Citrobacter koseri* to *Escherichia coli*) and shared just one species, the *Lactobacillus jensenii*.

Stable community structure types within individuals were observed in 4 (U3, U7, U11, U23) out of 10 individuals and were represented by *Lactobacillus crispatus, Bifidobacterium* spp. with *Lactobacillus paragasseri* or *Streptococcus agalactiae,* in combination with other Gram-positive bacteria.

Interestingly, two of those sample pairs (U7 and U23) are from individuals that acquired UTI, followed by antibiotic treatment, in the interval between samplings. Maintenance of 9 species (23.7%) including *Lactobacillus paragasseri* was observed in U7 sample pair, and 10 species (41.7%), including *Lactobacillus crispatus* and *Gardnerella* genomospecies 3 was observed in U23 sample pair, despite changes in their relative abundance*.*

Additional analysis based on genus level revealed higher stability, with 6 out of 10 individuals comprising sample pairs in the same clusters. Overall, the highest number of samples belonged to the cluster represented by *Lactobacillus* genus, followed by clusters characterized by abundant *Gardnerella* or *Streptococcus* genera. Heatmap and dendrogram representing hierarchical clustering at genus level is available in Additional file 2: Fig. S2 and Fig. S3, respectively.

## Discussion

In this study, using a comprehensive and extended culturomic approach, we enlarged the knowledge on diversity of FUM and its bacterial community structures in asymptomatic individuals. We also demonstrated FUM stability in two timepoints within long period of time.

Most of FUM studies are based on genus level classification and on most dominant taxa [12–16], while particular functional characteristics are often species specific, e.g., antimicrobials or metabolites production [21, 22].

In our study, due to the higher number of morphotypes characterized per sample and the higher taxonomic resolution conferred by genotypic markers used (e.g, *pheS*), we captured a slightly higher amount of species, however reflecting a similar diversity as previous reports [2, 12]. For instance, *Gardnerella swidsinskii* and *Gardnerella* genomospecies 3 were here identified for the first time within urogenital microbiota of asymptomatic individuals, in addition to *Gardnerella vaginalis*, suggesting their frequent occurrence in healthy FUM. Moreover, higher species diversity within Lactobacillaceae was captured with the recently described *Lactobacillus mulieris* infrequently observed among the samples tested [20].

Although alpha diversity measures (mean Shannon index < 1.5) suggest that FUM is less diverse than other human body niches [17], we observed a high overall species variability and diverse community structure types. Hierarchical community clustering based on Bray-Curtis dissimilarity matrix enabled to capture various community structure types based on bacterial species combinations. The largest cluster was characterized by the commonly described *Lactobacillus crispatus*, often in combination with *Gardnerella vaginalis* or *Gardnerella* genomospecies 3. *Gardnerella* species were also observed in other community structure types, usually with only one species present in individual FUM, confirming previously reported high occurrence of this genus in urinary microbiota [16, 17]. The remaining community structure types were characterized by many diverse species, including species commonly associated with UTI.

Of interest, FUM composition differences, related to *Finegoldia magna* and *Streptococcus anginosus,* according to smoking status were observed, requiring further validation. Those species were previously associated with urinary symptoms presence and/or severity [17, 23].

Within individuals, FUM changes were frequently detected at two distant timepoints (e.g., *Gardnerella swidsinskii, Atopobium vaginae and Dialister microaerophilus* community type converted to *Gardnerella vaginalis*, *Bifidobacterium* spp., *Cutibacterium avidum* type). This data suggests, the possibility of interchange between certain bacterial groups that might share common metabolic functions. Additionally, few communities that maintained their composition at two timepoints were detected and characterized by combinations of *Lactobacillus crispatus, Bifidobacterium* spp. with *Lactobacillus paragasseri* or *Streptoccocus agalactiae*. Further evidence on the resilience of those communities is the maintenance of *Lactobacillus crispatus* or *Bifidobacterium* spp. with *Lactobacillus paragasseri* community structure type in women after antibiotic treatment for a UTI. Studying short-term FUM dynamics, Price et al. also reported resilience of lower urinary tract microbiota in communities with dominance of e.g., *Lactobacillus* or *Lactobacillus* and *Gardnerella* combination [14]. Similarly to their findings, at the genus level, changes in relative abundance of *Lactobacillus*, *Gardnerella* or *Streptococcus* were observed in three individuals, leading to change in community structure type [14].

Moreover, maintenance of *Gardnerella* species, *Lactobacillus gasseri* and *Lactobacillus jensenii* was also observed in certain sample pairs, for which different FUM composition was observed at two time points tested. The protective role of particular strains belonging to *Lactobacillus jensenii* [24, 25]*,* can possibly contribute to the health maintenance of an individual with high abundance of Enterobacteriaceae*,* including an uropathogenic ST131 *Escherichia coli* (UPEC) according to its gene content [26] (unpublished data). This data also highlights the relevance of strain level characterization to understand FUM role in health and disease, as previously noticed [27, 28].

It is notable that our study focused on the group of well characterized young age European women, contrary to most urinary and urogenital microbiota studies [4, 6, 9–12, 17]. Moreover, samples were collected only on the 3rd week of women’s menstrual cycle to prevent interferences (e.g., menstrual discharge), which was recently demonstrated by Price et al., [14] as factor influencing microbiota composition. Women providing samples over time were under similar lifestyle and physiological conditions.

Although our cohort included a small number of participants, we believe that detailed description of a small group of women may substantially enlarge knowledge originated from studies with large scale cohorts but less detailed analysis. Additionally, we are aware that our choice of using voided urine samples brings a risk of genital contamination, thus representing more accurately the urogenital microbiome. However, Chen et al., recently reported that prevalence of *Lactobacillus* and *Gardnerella* were detected with equal sensitivity in voided urine and urine collected by catheter [15]. Moreover, we are convinced that characterizing microbiota from samples routinely used for screening and diagnosis is highly valuable to facilitate accurate results interpretation and potentiate their use in future diagnostics. Undoubtedly, knowing also genital tract microbiota composition would be highly beneficial to enlarge our understanding of health-associated and pathogenic strains similarity between urinary and vaginal microbiota [28].

Another potential limitation could be the lack of culture-independent DNA-based data, however current diagnostic procedures for urine samples are based on culturing. Moreover, culturomic approach is necessary to assess alive bacterial communities and provide isolates for further characterization.

## Conclusions

In this study, we characterized species level stability of the FUM of reproductive age women at two timepoints within a long period of time. We present further evidence that FUM can be dynamic over time and multiple FUM communities may be associated with urogenital tract of some asymptomatic individuals.

Additionally, at 2 sampling points with long time interval, we identified community structure types that seem to indicate persistence of certain species in healthy FUM and provides further evidence on resilient bacterial communities.

We also revealed previously unknown diverse community structure types in healthy FUM. These findings may challenge further identification of eubiotic and dysbiotic states and consequently, diagnostic and treatment strategies for urogenital and urinary tract pathologies.

Moreover, our results support that culturomic analysis with the large-scale isolates characterization is a valuable tool for microbiota diversity description and provides isolates for further analysis.

The future studies focusing on strain level characterization to discern functions contributing to health maintenance in urogenital tract are required. Furthermore, healthy FUM structures characterized by highly abundant species commonly associated with UTI, as here reported, highlight the need for a better understanding of microbiota-host interactions.

## Methods

### Participant information

Ten asymptomatic women (24–40 years old) were recruited to voluntarily participate in the FUM study conducted at the Faculty of Pharmacy, University of Porto, Portugal, at two time points. All women provided informed written consent for participation in the study and fulfilled a detailed questionnaire containing demographic, health-associated and lifestyle information before both sampling times. The study was developed according to the Helsinki Declaration principles and the protocol was submitted and approved by the Ethical Commission of Faculty of Pharmacy, University of Porto. Inclusion criteria at both sampling times were no pregnancy, no antibiotic treatment in the previous month and no current symptoms or diagnosis of urinary tract infection (UTI).

### Sample collection

Ten women provided first morning voided midstream urine samples at two time points (total number of samples = 20; sample pairs = 10). Interval between first and second sample collection varied in a range of 11 and 28 months, depending on donors’ availability. Samples were collected in the 3rd week of the menstrual cycle. Participants also provided vaginal swab collected prior to urine sample collection (data not shown). Detailed verbal and written instructions were provided to each woman before sampling. Flyer included written and graphical information on proper wash prior to sampling and vaginal swab collection in order to minimize possible vulvo-vaginal contamination.

### Sample analysis

Urine samples were subjected to an extended culturomic analysis within 2 h from sample collection. The extended culturomic protocol is a modification of the expanded quantitative urine culture (EQUC) previously described [2]. Culture included plating of 100 μl of urine into 140 mm diameter-Petri dishes. Protocol included Columbia Agar with 5% sheep blood (Biogerm, Portugal) and chromogenic agar typically used for uropathogens detection (HiCrome UTI, HiMedia, India) supplemented with previously described nutrients i.e., 2% (w/v) gelatin, 0.5% (w/v) yeast extract, 0.1% (w/v) starch, 0.1% (w/v) glucose and 0.1% (v/v) Tween 80 [29, 30]. Incubation at 37 °C for 48 h was performed under aerobic, microaerophilic, and anaerobic atmospheric conditions for Columbia Agar plates, and aerobic and microaerophilic condition for supplemented chromogenic agar (GENbox MICROAER and GENbox ANAER, bioMérieux, France). Besides culture, dipstick test (Combur-Test, Roche) and microscopy examination were performed. Additionally, when during microscopic examination a higher bacterial load was suspected, diluted volume of urine was plated and incubated following the same protocol. Each colony morphotype was quantified to obtain a most approximate number of colony forming units per milliliter (CFU/ml) and up to 5 colonies of the same morphotype were isolated, stored and subjected to identification. Multiple representatives were isolated to ensure reliable microbiota profiling. Many species belonging to genera widely present within urogenital microbiota e.g., *Lactobacillus*, *Staphylococcus*, *Corynebacterium* have very similar or equal colony morphology, thus their diversity may be easily underestimated.

### Isolates identification

Firstly, all isolates were subjected to matrix-assisted laser desorption/ionization time-of-flight mass spectrometry (MALDI-TOF MS) VITEK MS system (bioMérieux, France), using in-vitro diagnostic database version 3.0. In case of no identification by MALDI-TOF MS isolates were subjected to 16S rRNA gene sequencing and/or other suitable genotypic biomarkers (*pheS*, *rpoB, recN*) [31–34]. Additionally, due to recent taxonomic reclassification of genus *Gardnerella*, all isolates identified as *Gardnerella vaginalis* by MALDI-TOF MS were subjected to *cpn60* gene sequencing [35, 36].

### Statistical analysis

Continuous and categorical variables referring to participants’ demographic and lifestyle characteristics were interpreted based on descriptive statistics. All community analyses were based on relative abundance (%) calculated as the CFU percentage of identified species from total CFU/ml count. Alpha-diversity (within-samples diversity) represented by the number of observed species and Shannon index (increases when species richness and evenness increase), was performed and visualized using phyloseq package (version 1.30.0) [37] R version 3.6.1 [38]. Pielou’s evenness index was calculated; evenness refers to the distribution of species in terms of relative abundance. Pielou’s index comprises values between 0 and 1, where lower values stand for lower degree of evenness. Figure representing cumulative relative abundance of shared species was created with phyloseq package. Beta-diversity (between-samples diversity) was represented by Bray-Curtis dissimilarity matrix with values comprised between 0 and 1, where 0 states for high similarity and 1 for high dissimilarity, and 2-dimension Non-metric Multi-dimensional Scaling (NMDS) with samples ordination based on dissimilarity matrix. Stress value was measured using vegan (version 2.5.6) [39] package. Heatmap representing Bray-Curtis dissimilarity matrix was generated with lattice package (version 0.20.38) [40]. NMDS plot was performed using phyloseq package. Statistical significance for age, body mass index, smoking status, previous UTI, usage of anti-inflammatory drugs in a week before sampling, hormonal contraceptives usage, previous pregnancies and presence or absence of menstrual cycle was accessed with analysis of similarities (ANOSIM) performed with vegan package, based on Bray-Curtis dissimilarity matrix. ANOSIM analysis result in significance level (*p* value) and R value where number close to 0 stands for similarity, and close to 1 stand for dissimilarity. Multilevel pattern analysis for identification of bacterial species responsible for community divergence was accessed using indicspecies package (version 1.7.9) [41]. A heatmap including a dendrogram for hierarchical clustering with cutoff value of 0.8 to define the clusters was generated using vegan (version 2.5.6) and gplots (version 3.0.1.2) [42] R packages. Hierarchical clustering was performed using unweighted pair group method with arithmetic mean (UPGMA) based on Bray-Curtis dissimilarity matrix. Additionally, ggplot2 (version 3.2.1) package [43] was used for data visualization.

## Supplementary Information

**Additional file 1: Table S1.** Species identified in each participant during first and second sampling and their relative abundance (%). **Table S2.** Number of observed species, Pielou’s evenness index and Shannon index per each sample.

**Additional file 2: Figure S1.** Dendrogram representing samples hierarchical clustering based on species level identification. A cutoff value of 0.8 was used to define the clusters (dashed blue line). **Figure S2.** Heatmap based on abundance (%) of genera detected. Dendrogram presents clustering of microbiota profiles into community structure types, based on 0.8 cutoff. Colorful bar below the dendrogram stands for different community structure types. **Figure S3.** Dendrogram representing samples hierarchical clustering based on genus level identification.

## Data Availability

The raw datasets generated and analyzed during the current study and accession numbers for DNA sequences deposited in NCBI database are available in the GitHub repository (https://github.com/magksi/FUM_stability.git).

## Abbreviations

FUM: Female urogenital microbiota
UTI: Urinary tract infection
CFU: Colony forming units
MALDI-TOF MS: Matrix-assisted laser desorption/ionization time-of-flight mass spectrometry
NMDS: Non-metric Multi-dimensional Scaling
SD: Standard deviation
ANOSIM: Analysis of similarities
