# Supplementary material for: Long-term stability of the urogenital microbiota of asymptomatic European women
Source: BMC Microbiol. 2021 Feb 25;21:64. doi: 10.1186/s12866-021-02123-3 (PMC7905919; doi:10.1186/s12866-021-02123-3)
Supplement: Supplementary file 2 — Additional file 2: Figure S1. Dendrogram representing samples hierarchical clustering based on species level identification. A cutoff value of 0.8 was used to define the clusters (dashed blue line). Figure S2. Heatmap based on abundance (%) of genera detected. Dendrogram presents clustering of microbiota profiles into community structure types, based on 0.8 cutoff. Colorful bar below the dendrogram stands for different community structure types. Figure S3. Dendrogram representing samples hierarchical clustering based on genus level identification. [file 12866_2021_2123_MOESM2_ESM.docx]

**Additional file 2. Supplementary Figures S1-S3.**

**Long-term stability of the urogenital microbiota of asymptomatic European women**

Magdalena Ksiezarek^1^, Svetlana Ugarcina-Perovic^1^, Joana Rocha^1^, Filipa Grosso^1^, Luísa Peixe^1^.

^1^UCIBIO-REQUIMTE. Laboratory of Microbiology, Faculty of Pharmacy, University of Porto, 4050 Porto, Portugal


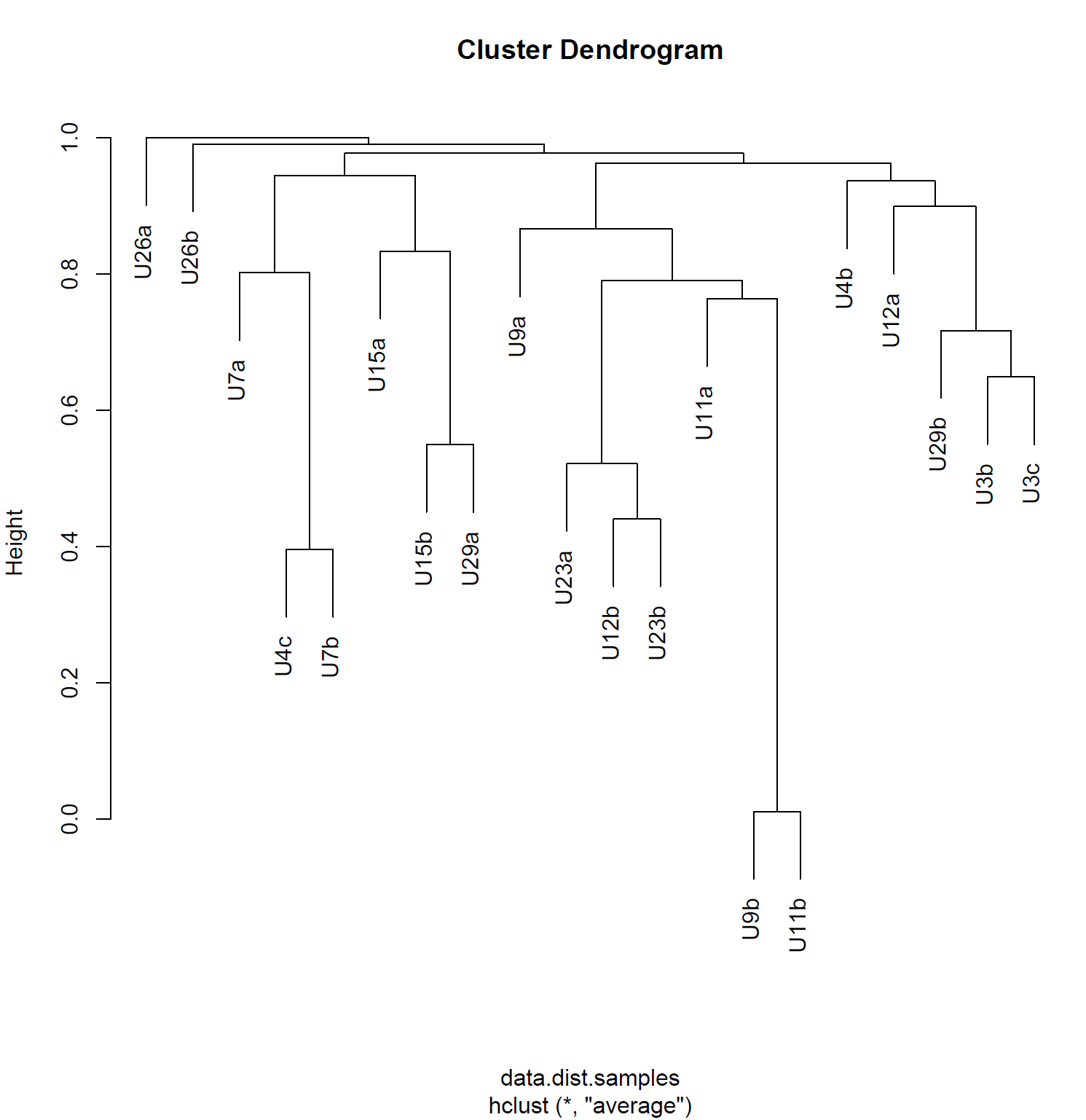


**Supplementary Figure S1.** Dendrogram representing samples hierarchical clustering based on species level identification. A cutoff value of 0.8 was used to define the clusters (dashed blue line).


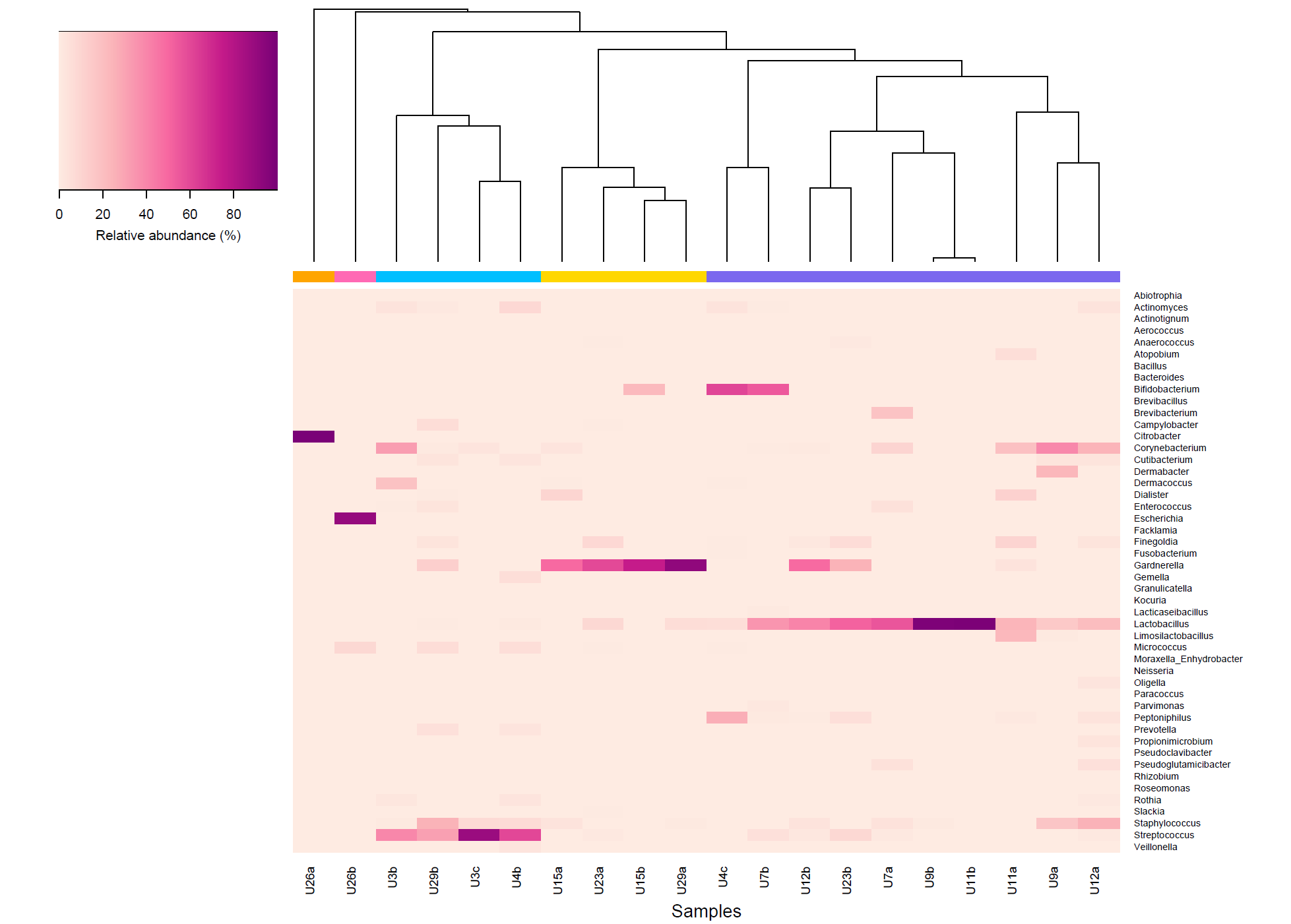


**Supplementary Figure S2.** Heatmap based on abundance (%) of genera detected. Dendrogram presents clustering of microbiota profiles into community structure types, based on 0.8 cutoff. Colorful bar below the dendrogram stands for different community structure types.


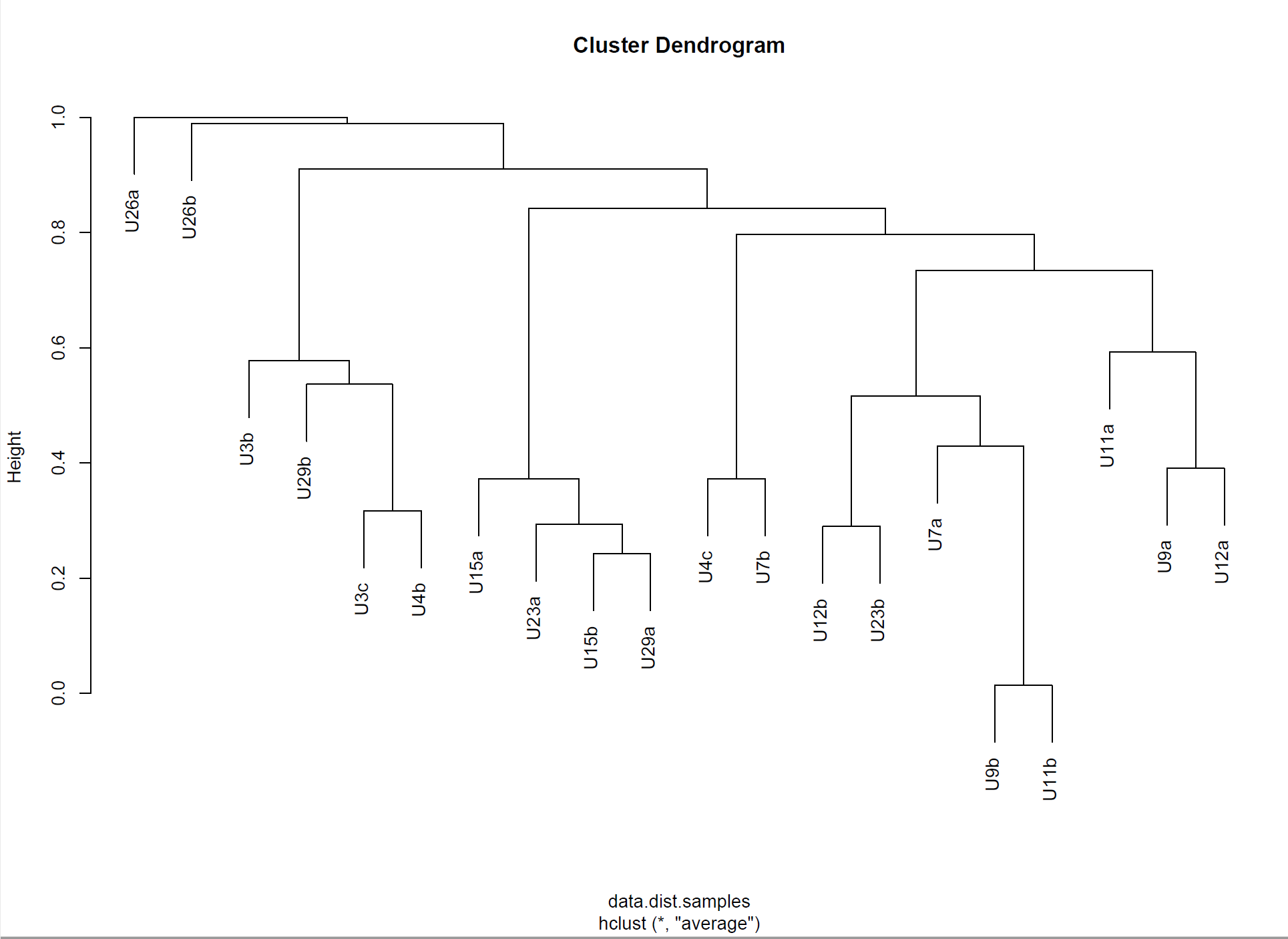


**Supplementary Figure S3.** Dendrogram representing samples hierarchical clustering based on genus level identification.
